# An Ayurvedic herbal extract inhibits oral epithelial cell IL-8 responses to host and bacterial agonists

**DOI:** 10.1186/s12906-020-2850-8

**Published:** 2020-02-27

**Authors:** Ana M. Chang, Shatha Bamashmous, Richard P. Darveau, Sunethra Rajapakse

**Affiliations:** 1 Department of Oral Health Sciences, University of Washington School of Dentistry, Seattle, WA USA; 2 Division of Periodontics, Farculty of Dentistry, King Abdulaziz University, Jeddah, Saudi Arabia; 3 Department of Periodontics, University of Washington School of Dentistry, Seattle, WA USA; 4 Department of Oral Medicine and Periodontology, University of Peradeniya, Peradeniya, Sri Lanka

**Keywords:** Herbal, Interleukin-8, Periodontitis, Oral health, *Fusobacterium nucleatum*, Inflammation

## Abstract

**Background:**

Natural products constitute a promising class of therapeutics for the treatment of gingivitis and periodontitis as well as the maintenance of oral health. However, the limited understanding behind their potential mechanisms and modes of action have hampered their incorporation into popular western therapeutics. This in vitro study characterizes an Ayurvedic herbal extract mixture, which has been clinically shown to promote gingival health and homeostasis.

**Methods:**

Telomerase immortalized gingival keratinocytes (TIGK) were infected with either *Fusobacterium nucleatum* cell wall, live *F. nucleatum*, IL-1β or TNF-α for 4 hours with and without the herbal extract. The immunomodulatory effects of the extract on host IL-8 production was measured by ELISA.

**Results:**

It was found that the Ayurvedic herbal extract mixture inhibited gingival epithelial cell IL-8 expression in response to both bacterial and host cytokine agonists. The herbal extract inhibited IL-8 stimulated by *F. nucleatum* cell wall, live *F. nucleatum*, IL-1β, and TNF-α in a dose-dependent manner that was not a result of host cell death. Furthermore, the extract showed significantly different ID_50_ doses demonstrating the differential ability to modulate both stimulated and basal IL-8 levels.

**Conclusions:**

In vitro investigation of this herbal extract mixture revealed that it has the ability to modulate gingival epithelial cell IL-8 expression in response to stimulation by bacterial components and host pro-inflammatory signals. This data demonstrates that the reduction in the gingival epithelial cell IL-8 response may in part be responsible for the previously reported ability of the Ayurvedic herbal extract mixture to reduce gingivitis in two separate human clinical studies.

## One-sentence summary

An ayurvedic herbal extract modulates epithelial cell IL-8 expression, a key host defense component in oral health and disease.

## Background

Maintenance of oral health is the most effective strategy for prevention of disease. Irremediable disturbances to this carefully intertwined homeostatic state between the host inflammatory response and the oral microbial community can ultimately lead to disease and is characterized by dysbiosis of both the microbial community and the host immune response [1, 2]. Currently, inadequate understanding of the complex inflammatory networks modulated in both oral health and disease has limited the number of non-invasive therapeutic or maintenance approaches available which reduce the occurrence or severity of either gingivitis or periodontitis [3, 4]. Therefore, there is a need to identify novel therapeutic interventions and oral maintenance programs to address oral health. Naturopathic medicine potentially represents one area where new oral health regimes may prove to be beneficial. Numerous studies have identified plant extracts that possess potent antibacterial, antifungal, and anti-inflammatory qualities, which inhibit key inflammatory mediators and have been in use for centuries to maintain oral hygiene [5–11].

For example, in Sri Lanka, a time tested and proprietary Ayurvedic recipe of blended plant extracts has been incorporated into a toothpaste (Sudantha^1^). The plants used in this product are: heartwood of cutch tree (*Acacia chundra* Willd.), malabar nut leaf (*Adhatoda vasica* Nees.), Spanish cherry bark (*Mimusops elengi* L.), black pepper (*Piper nigrum* L.), pongam oil tree root (*Pongamia pinnata*(L.) Pirerre), Aleppo oak galls (*Quercus infectoria* Olivier.), clove (*Syzygium aromaticum* L.), myrobalan fruit (*Terminalia chebula* Retz.), and ginger (*Zingiber officinale* Roscoe) and have been used traditionally in Ayurveda for oral care [12–18]. This product has been examined in two separate randomized double-blind placebo-controlled clinical trials. In one human trial, the effects of this herbal extract on oral hygiene and gingival health showed significant reduction of gingival bleeding, dental plaque formation, and salivary anaerobic bacterial counts as early as 4 weeks of its use compared to the placebo group [19]. Moreover a follow-up clinical trial investigating these therapeutic benefits for patients with gingivitis confirmed these results, reporting a reduction in gingival bleeding, plaque score, total salivary anaerobic bacterial counts, and probing pocket depth [9]. Altogether, these randomized clinical studies provide robust evidence of the effective antiplaque and anti-gingivitis effects of this herbal extract for both the maintenance of health and treatment of disease.

Interleukin-8 (IL-8) is a key inflammatory mediator involved in chemotaxis [20] and activation [21] of immune cells, such as neutrophils, as well as promotion of tissue remodeling and angiogenesis [22]. In humans, gingival keratinocytes have been shown to express IL-8 in response to oral bacteria [23], including the “bridging” organism *Fusobacterium nucleatum* [24, 25], and pro-inflammatory cytokines IL-1β [26] and TNF-α [27]. The modulation of IL-8 secretion in gingival epithelial tissues during episodes of periodontitis [28, 29] and gingivitis [30, 31] is considered a key component for the maintenance of oral health [1, 32]. Therefore, in order to elucidate potential mechanisms by which the medicinal extract Sudantha (SUD) contributes to the promotion of gingival health and homeostasis, its immunomodulatory effects on gingival epithelial cell IL-8 production was determined.

This study found that SUD inhibited expression of the pro-inflammatory cytokine, IL-8, by gingival epithelial cells agonized with bacterial products (*F. nucleatum* cell wall extracts or live *F. nucleatum*) and host inflammatory mediators (IL-1β and TNF-α) in a dose-dependent manner. These data support the notion that one aspect of the efficacy of the Sudantha extract is in its ability to reduce excessive IL-8 secretion in response to both bacterial and host inflammatory signals.

## Methods

### Bacterial culture and crude cell wall

*F. nucleatum* ATCC 25586 was obtained from the Darveau laboratory bacterial collection and grown overnight in trypticase soy yeast broth (TYK) supplemented with 10 μg/mL hemin and 1 mg/mL menadione at 37 °C under anaerobic conditions (80% N2, 10% CO2, 10% H2). *F. nucleatum* crude cell wall samples were prepared as previously described [33] using a French cell pressure of 15,000 lb./in^2^.

### TIGK cell culture and infection

The immortalized human gingival keratinocyte cell line, TIGK, was generously provided by Dr. Richard J. Lamont^2^ and maintained in growth medium^3^ containing 25 μg/mL bovine pituitary extract, 0.2 ng/mL human recombinant epidermal growth factor, 0.4 mM calcium chloride, and 10% penicillin-streptomycin. Antibiotics were excluded for experiments with live bacteria.

TIGK cells were plated into 96-well plates at a density of 2 × 10^4^ cells/well and allowed to grow for 48 h until a confluence of approximately 90%. Test wells were stimulated in triplicate for 4 h with or without the extract (controls) at the indicated concentrations at 37 °C and 5% CO_2_ with the indicated ligands: live *F. nucleatum* bacteria at a multiplicity of infection (MOI) of 1:500 and *F. nucleatum* cell wall components, IL-1β⌷,^4^ and TNF-α^5^ all at 100 ng/ml.

### Sudantha herbal extract

Sudantha (SUD) extract, provided by Dr. Devapriya Nugawela,^6^ is a crude dark proprietary mixture of herbs that is incorporated into a commercially available toothpaste^††^. The formula of SUD is based on the recommendation of a specialist panel of Ayurvedic clinicians and contains a mixture of heartwood of cutch tree (*Acacia chundra* Willd.), malabar nut leaf (*Adhatoda vasica* Nees.), Spanish cherry bark (*Mimusops elengi* L.), black pepper (*Piper nigrum* L.), pongam oil tree root (*Pongamia pinnata*(L.) Pirerre), Aleppo oak galls (*Quercus infectoria* Olivier.), clove (*Syzygium aromaticum* L.), myrobalan fruit (*Terminalia chebula* Retz.), and ginger (*Zingiber officinale* Roscoe). SUD is standardized and quality controlled by high performance liquid chromatography (HPLC). It was stored at 4 °C in the dark and freshly prepared to a stock concentration of 2 mg/mL in 0.2% ethanol. This stock concentration was then subsequently serially diluted two fold with TIGK growth medium to produce working concentrations of 250 μg/mL, 125 μg/mL, 62.5 μg/mL, 31.25 μg/mL, 15.6 μg/mL, 7.8 μg/mL, 3.9 μg/mL, and 1.95 μg/mL.

### Measurement of secreted IL-8 by enzyme-linked immunosorbent assay (ELISA)

After termination of the 4 h infection, culture supernatants were collected and diluted 2.5 fold in 1% bovine serum albumin in 1X PBS for determination of secreted IL-8 by standard sandwich ELISA. IL-8 monoclonal primary capture antibody^7^ and secondary biotin-labeled, detection-antibody^8^ were used for ELISA and detected with avidin-horseradish peroxidase enzyme (HRP) and tetramethylbenzidine (TMB) substrate. Optical densities were read at 450-570 nm on a microplate reader^9^ and concentrations were calculated from a standard curve using known concentrations of serially diluted recombinant human IL-8.^10^

### Half-maximal inhibitory dose (ID_50_)

Half-maximal inhibitory dose (ID_50_) was estimated^11^ from an experimentally derived dose-response curve for each concentration.

### Cell viability

After removal of supernatant for IL-8 protein determination, cell viability was assessed using a fluorometric assay^12^ according to manufacturer protocols. In brief, 50 μl of growth medium was added to cells and followed by the addition of 50 μl of fluorometric reagent. Luminescence was measured after 10 min at room temperature using a microplate luminometer.^13^ Cell viability was assayed with each experiment.

### Statistical analysis

Student t tests were performed^§§§§^ to determine significance of IL-8 responses by stimulated TIGK cells with and without extract. *P* values below 0.05 was considered significant (* *P* ≤ 0.05 ** *P* ≤ 0.01, *** *P* ≤ 0.001).

## Results

### Concentrations equal to or less than 250 μg/ml of SUD does not affect TIGK cell viability

**Fig. 1:**
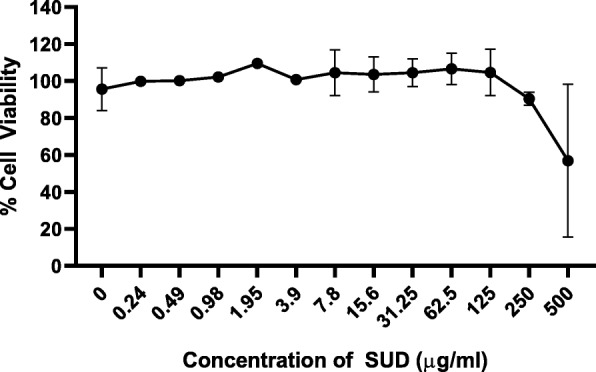
Concentrations equal to or less than 250 μg/mL of SUD does not affect TIGK cell viability. Percent cell viability output of TIGK cells after 4 h incubation with SUD. Error bars represent standard deviations for experiments with concentrations at 0 and above 7.8 μg/ml which were performed three independent times in triplicate

The effect of SUD on TIGK cell viability was measured to find the optimal concentrations for further downstream experimentation. Exposure to SUD for 4 h revealed that concentrations equal to or less than 250 μg/ml did not affect TIGK cell viability (Fig. 1). In contrast, concentrations of 500 μg/ml SUD showed cytotoxicity, reducing TIGK cell viability to 62%, and was excluded from further experimentation. Therefore, downstream characterization on the effects of SUD on modulation of host inflammatory mediators were performed with 250 μg/ml as the maximal dose. Furthermore, cell viability was examined concurrently with each experiment and showed similar results with no effect on TIGK cell viability.

### SUD suppresses F. nucleatum cell wall extract induced IL-8 expression by gingival keratinocytes

**Fig. 2:**
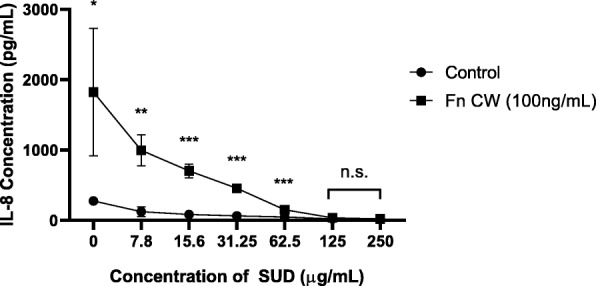
SUD suppresses *F. nucleatum* cell wall induced IL-8 expression by gingival keratinocytes. A representative figure of IL-8 expression measured by ELISA of TIGK cells infected for 4 h by *F. nucleatum* cell wall (100 ng/mL) with SUD performed three independent times in triplicate. Controls are cell culture medium with the indicated concentrations of SUD. Error bars represent standard deviations. Significant statistical differences were calculated using the student t-test (* *P* ≤ 0.05, ** *P* ≤ 0.01, *** *P* ≤ 0.001, n.s. not significant). No statistical significance indicates IL-8 levels were significantly reduced similar to basal levels

*F. nucleatum* represents a common Gram negative species found in gingival plaque obtained from periodontally healthy and diseased [34–37] sites and have been shown to elicit a potent IL-8 response from gingival epithelial cells [24, 25, 38]. TIGK cells infected with 100 ng/ml of *F. nucleatum* cell wall extracts produced a potent IL-8 response that was suppressed by the addition of SUD in a dose dependent manner and that was not a result of cell death (Fig. 2). Suppression of IL-8 by *F. nucleatum* cell wall was first observed at 7.8 μg/ml SUD, while SUD at a range of 125–250 μg/ml worked optimally to reduce IL-8 expression down to basal levels without affecting cell viability.

### SUD suppresses live F. nucleatum-induced IL-8 expression by gingival keratinocytes

**Fig. 3:**
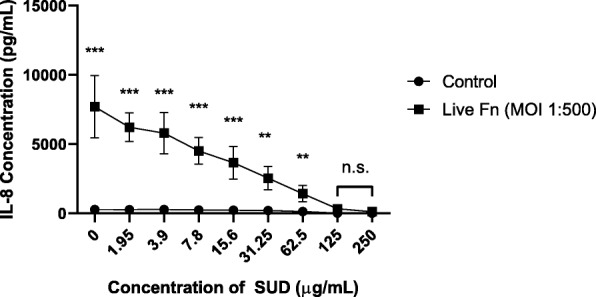
SUD suppresses live *F. nucleatum* induced IL-8 expression by gingival keratinocytes. A representative figure of IL-8 expression measured by ELISA of TIGK cells infected for 4 h by live *F. nucleatum* (MOI 1:500) with SUD performed four to five independent times in triplicate. Controls are cell culture medium with the indicated concentrations of SUD. Error bars represent standard deviations. Significant statistical differences were calculated using the student t-test (** *P* ≤ 0.01, *** *P* ≤ 0.001, n.s. not significant). No statistical significance indicates IL-8 levels were significantly reduced similar to basal levels

To further characterize the anti-inflammatory effects of SUD against *F. nucleatum* induced IL-8 production, TIGK cells were infected with live bacteria at an MOI of 1:500. Similar to bacterial cell wall, live *F. nucleatum* at an MOI of 1:500, produced a potent IL-8 response (Fig. 3) greater than that observed with *F. nucleatum* cell wall alone and its expression was similarly dampened by SUD at concentrations of 1.95 μg/ml. This inhibition occurred in a dose dependent manner that brought IL-8 expression to basal levels of IL-8 with 125–250 μg/ml of SUD, similar to concentrations required to reduce IL-8 levels produced by *F. nucleatum* cell wall. Despite observable trends in IL-8 reductions at all test SUD concentrations, student t test confirmed the loss of statistical significance at 125–250 μg/ml of SUD, indicating significant reduction of IL-8 levels down to basal levels.

### SUD suppresses IL-1β and TNF-α host mediator induced IL-8 expression by gingival keratinocytes

**Fig. 4:**
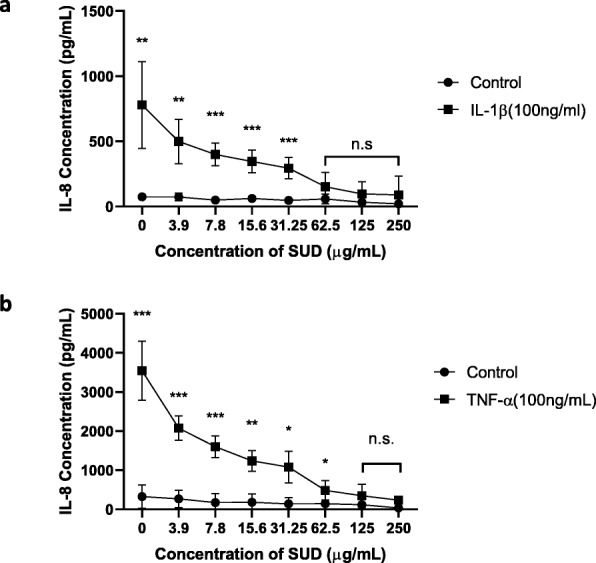
SUD suppresses IL-1β and TNF-α host mediator induced IL-8 expression by gingival keratinocytes. a. Representative figures of IL-8 expression measured by ELISA of TIGK cells infected for 4 h with exogenous IL-1β and b. TNF-α at 100 ng/ml performed three independent times in triplicate. Controls are cell culture medium with the indicated concentrations of SUD. Error bars represent standard deviations of experiments. Significant statistical differences were calculated using the student t-test (* *P* ≤ 0.05, ** *P* ≤ 0.01, *** *P* ≤ 0.001, n.s. not significant). No statistical significance indicates IL-8 levels were significantly reduced similar to basal levels

IL-1β and TNF-α are potent inflammatory cytokines reported to be involved in cellular proliferation, activation, and differentiation [39]. These host cytokines have been shown to induce IL-8 secretion from gingival epithelial cells [26, 27, 40, 41]. Consistent with previous reports of IL-1β and TNF-α induced expression of IL-8 by gingival keratinocytes, TIGK cells infected with 100 ng/ml of each cytokine for 4 h induced expression of IL-8 to 779 pg/ml and 3546 pg/ml, respectively (Fig. 4a and b). Inhibition of IL-8 occurred with the addition of SUD in a dose-dependent manner that was able to bring down IL-8 expression induced by IL-1β to 89 pg/ml and TNF- α induced expression to 235 pg/ml, almost down to control levels of 21–36 pg/ml IL-8. Statistical analysis revealed loss of statistical significance at concentrations of 62.5–250 μg/ml for IL-1β and 125–250 μg/ml for TNF- α.

### Differential inhibition of agonist stimulated IL-8 expression by gingival keratinocytes

**Fig. 5:**
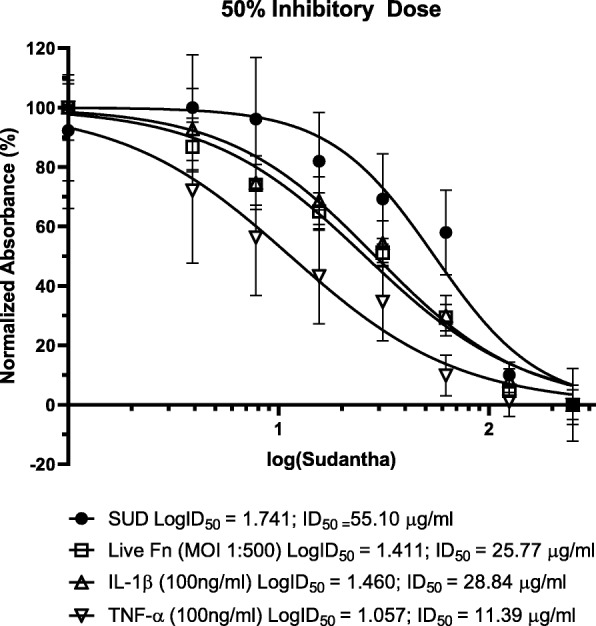
Differential inhibition of agonist stimulated IL-8 by gingival keratinocytes. Fifty percent inhibitory dose (ID50) estimated from an experimentally derived dose-response curve for each concentration of Sudantha (SUD) against basal levels of IL-8 (SUD Control) and IL-8 induction by live F. nucleatum (MOI 1:500), IL-1, and TNF-α (both at 100 ng/ml) performed three independent times. Controls are cell culture medium with the indicated concentrations of SUD. Error bars represent standard error of means

SUD was able to dampen both *F. nucleatum* and host cytokine induced gingival epithelial IL-8 inflammatory responses. Therefore, the 50% inhibitory dose (ID_50_), the concentration of the test compound required to inhibit the agonist induced cytopathogenic effect by 50% [42], was examined for preferential inhibitory effects between stimulation by bacterial products and host pro-inflammatory signals. Gingival epithelial cells secrete basal levels of IL-8 (Fig. 5) which was dampened to 50% expression by SUD at concentrations of 55.10 μg/ml. In contrast to mechanisms related to basal expression of IL-8, agonist stimulated IL-8 was dampened to 50% inhibitory levels at a much lower concentration. TNF-α stimulated IL-8 response was most sensitive to SUD with ID_50_ concentrations at 11.39 μg/ml SUD. While, IL-1 and live *F. nucleatum* required higher ID_50_ concentrations around 28.84 μg/ml and 25.77 μg/ml SUD respectfully. Therefore, these ID_50_ results demonstrate differential inhibitory effects of the extract between different IL-8 agonists.

## Discussion

In general, the goal of treatment is to aid and enhance the inherent ability of the host’s innate defense mechanisms to restore compromised homeostasis. Consistent with this approach, the use of plant derived polyphenols as anti-inflammatory compounds have been intensely investigated [5–11] and shown to inhibit key mediators of the inflammatory cascade, including MAP kinases and nuclear transcription factors [5]. SUD, a proprietary mixture of natural herbs, has recently shown clinical success in its ability to restore and maintain gingival and periodontal health while providing antimicrobial activity [9, 19]. This manuscript describes the anti-inflammatory mechanisms exhibited by SUD against bacterial or host cytokine induced gingival epithelial cell IL-8 secretion and the potential benefits of reducing IL-8 in therapeutics.

IL-8 is a pro-inflammatory cytokine produced by a wide variety of cells including gingival epithelial cells, endothelial cells, gingival fibroblasts, neutrophils, monocytes, and phagocytes in response to bacterial invasion and plays a distinct role in neutrophil migration and activation [23, 39]. The importance of its role in neutrophil function has been recognized in oral health and disease [43, 44]. Irregular and uncontrolled expression of IL-8 contributes to neutrophil mediated-local-tissue-destruction (bystander damage) of periodontal tissues [1, 44]. Therefore, therapeutic approaches targeted towards the regulation of IL-8, and hence neutrophil homeostasis, would be greatly beneficial. Our study showed that TIGK cells stimulated with either live *F. nucleatum* or its cell wall components produced a potent IL-8 response which was suppressed by the addition of SUD in a dose dependent manner without affecting cell viability. Although there was reduction of IL-8 with the addition of SUD at all concentrations tested, significant reduction was shown at concentrations of 125–250 μg/ml when compared between SUD and control samples.

IL-1β and TNF-α are potent pro-inflammatory mediators secreted in response to bacteria and are associated with the pathogenesis and progression of periodontal disease [45–47]. They induce the upregulation of adhesion molecules on neutrophils and endothelial cells, stimulate the production of chemotactic molecules to induce neutrophil migration, and enhance inflammatory signals which potentiate inflammatory responses [48]. Consistent with this, gingival epithelial cells stimulated with IL-1β and TNF-α induced expression of IL-8 in this study, which was inhibited by SUD in a dose dependent manner. This inhibition was statistically significant at 62.5–250 μg/ml. Collectively, these data suggests that one mechanism behind the observed clinical efficacy of SUD in clinical trials may be due to its ability to dampen neutrophil migration through the reduction of host or bacterially mediated IL-8 secretion.

It is noteworthy that different concentrations of SUD was required to reduce IL-8 response half maximally after stimulation by IL-1β and TNF-α. TNF- α induced IL-8 required 11.39 μg/ml of SUD, while IL-1β induced IL-8 required 2-fold this amount, 28.84 μg/ml of SUD. Gingival keratinocytes are reported to produce varying levels of basal IL-8 secretion [32, 49] (which may be important in the maintenance of healthy homeostasis) and SUD was required in significantly higher concentrations, to dampen the basal levels of IL-8 expression when compared to the agonist activated IL-8 secretion. Differences in SUD inhibitory concentrations required to inhibit between basal and host inflammatory modulator stimulated IL-8 suggests that agents in SUD demonstrate selective inhibition of different IL-8 stimulation pathways which may prove to be a useful tool to modulate host inflammatory responses. However, further work is required to better understand the potential selective action of SUD on IL-8 secretion and the possible mechanisms behind its clinical success.

## Conclusions

Altogether, in vitro experiments of SUD on its ability to dampen the host immune response in relation to IL-8 stimulation by bacteria or host inflammatory mediators supports previously shown clinical beneficial effects of SUD for the maintenance of periodontal and gingival health. Specifically, this manuscript has demonstrated that at least one anti-inflammatory effect of SUD is the inhibition of gingival epithelial cell IL-8 secretion. Since, IL-8 is a potent neutrophil chemokine associated with gingivitis, the ability to dampen neutrophil migration represents a beneficial effect which may contribute to the efficacy observed in gingivitis clinical trials. However, additional experimentation is required to expand upon the potential of this extract to selectively modulate host inflammatory pathways without disturbing the cell intrinsic host inflammatory surveillance.

## Data Availability

All data generated or analyzed during this study are included in this published article.

## Abbreviations

HPLC: High performance liquid chromatography
HRP: Avidin-horseradish peroxidase enzyme
ID50: Half-maximal inhibitory dose
IL: Interleukin
MOI: Multiplicity of infection
SUD: Proprietary mixture of herbal extracts, Sudantha
TIGK: Telomerase immortalized gingival keratinocytes
TMB: Tetramethylbenzidine
TYK: Trypticase soy yeast broth

## Footnotes

1 Sudantha® (Reg.No.02/01/PV/08/143), Link Natural Products, Kapugoda, Sri Lanka

2 Department of Oral Immunology and Infectious Diseases, University of Louisville School of Dentistry, Kentucky, USA

3 Keratinocyte-SFM, Thermo Scientific, Waltham, Massachusetts, USA

4 Human IL-1β Recombinant Protein, eBioscience, San Diego, California, USA

5 Human TNF-α Recombinant Protein, eBioscience, San Diego, California, USA

6 Link Natural Products, Kapugoda, Sri Lanka

7 M801, Thermo Scientific, Waltham, Massachusetts, USA

8 M802B, Thermo Scientific, Waltham, Massachusetts, USA

9 VMax Microplate Reader, Molecular Devices, Sunnyvale, California, USA

10 Human IL-8 Recombinant Protein, eBioscience, San Diego, California, USA

11 GraphPad Prism 8, GraphPad Software, San Diego, California, USA

12 CellTiter-Glo, G9241, Promega, Madison, Wisconsin, USA

13 Centro LB 960, Berthold Technologies, Wildbad, Germany

## References

[1] Darveau RP (2010). Periodontitis: a polymicrobial disruption of host homeostasis. Nat Rev Microbiol.

[2] Marsh PD (2012). Contemporary perspective on plaque control. Br Dent J.

[3] Axelsson P, Nystrom B, Lindhe J (2004). The long-term effect of a plaque control program on tooth mortality, caries and periodontal disease in adults. Results after 30 years of maintenance. J Clin Periodontol.

[4] Chapple IL, Van der Weijden F, Doerfer C (2015). Primary prevention of periodontitis: managing gingivitis. J Clin Periodontol.

[5] Santangelo C, Vari R, Scazzocchio B, Di Benedetto R, Filesi C, Masella R (2007). Polyphenols, intracellular signalling and inflammation. Ann Ist Super Sanita.

[6] Khanna D, Sethi G, Ahn KS (2007). Natural products as a gold mine for arthritis treatment. Curr Opin Pharmacol.

[7] Zhang L, Zhao Y, Wang ZA (2016). The genus Boschniakia in China: an ethnopharmacological and phytochemical review. J Ethnopharmacol.

[8] Stoyell KA, Mappus JL, Gandhi MA (2016). Clinical efficacy of turmeric use in gingivitis: a comprehensive review. Complement Ther Clin Pract.

[9] Howshigan J, Perera K, Samita S, Rajapakse PS (2015). The effects of an Ayurvedic medicinal toothpaste on clinical, microbiological and oral hygiene parameters in patients with chronic gingivitis: a double-blind, randomised, placebo-controlled, parallel allocation clinical trial. Ceylon Med J.

[10] Casati MZ, Algayer C, Cardoso da Cruz G (2013). Resveratrol decreases periodontal breakdown and modulates local levels of cytokines during periodontitis in rats. J Periodontol.

[11] Correa MG, Pires PR, Ribeiro FV (2018). Systemic treatment with resveratrol reduces the progression of experimental periodontitis and arthritis in rats. PLoS One.

[12] Ministry of Health and Family Welfare, Department of Health, Govt. of India. The Ayurvedic pharmacopoeia of India. Part 1, Vol.1; 1989.

[13] Government of India, Ministry of Health and Family Welfare, Department of Ayurveda, Yoga & Naturopathy, Unani, Siddha and Homoeopathy. Ayurveda Pharmacopoeia. Vol.1 Part II; 1979.

[14] Government of India Minsitry of Health and Family Welfare Department of ISM & H. Ayurveda Pharmacopoeia. Vol I, Part III; 1985.

[15] Department of Ayurveda: Ministry of Health, Nutrition, and Indigenous Medicine. Bhavaprakasa. Vol I; 1981.

[16] Indian Drug Manufacturers' Association. Indian herbal pharmacopoeia. Vol I; 1998.

[17] Indian Drug Manufacturers' Association. Indian herbal pharmacopoeia. Vol II; 1999.

[18] Sri Lanka: Education Publication Department. Sushruta Samhita; 1962.

[19] Jayashankar S, Panagoda GJ, Amaratunga EA, Perera K, Rajapakse PS (2011). A randomised double-blind placebo-controlled study on the effects of a herbal toothpaste on gingival bleeding, oral hygiene and microbial variables. Ceylon Med J.

[20] Tonetti MS, Imboden MA, Lang NP (1998). Neutrophil migration into the gingival sulcus is associated with transepithelial gradients of interleukin-8 and ICAM-1. J Periodontol.

[21] Jones SA, Wolf M, Qin S, Mackay CR, Baggiolini M (1996). Different functions for the interleukin 8 receptors (IL-8R) of human neutrophil leukocytes: NADPH oxidase and phospholipase D are activated through IL-8R1 but not IL-8R2. Proc Natl Acad Sci U S A.

[22] Martin TA, Snyder CR, Jiang WG, Matsumoto K, Nakamura T (2001). Interleukin-8 and Angiogenesis. Growth Factors and their Receptors in Cancer Metastasis. vol. 2.

[23] Tonetti MS, Imboden MA, Gerber L, Lang NP, Laissue J, Mueller C (1994). Localized expression of mRNA for phagocyte-specific chemotactic cytokines in human periodontal infections. Infect Immun.

[24] Han YW, Shi WY, Huang GTJ (2000). Interactions between periodontal bacteria and human oral epithelial cells: Fusobacterium nucleatum adheres to and invades epithelial cells. Infect Immun.

[25] Peyret-Lacombe A, Brunel G, Watts M, Charveron M, Duplan H (2009). TLR2 sensing of F. nucleatum and S. sanguinis distinctly triggered gingival innate response. Cytokine.

[26] Eskan MA, Benakanakere MR, Rose BG (2008). Interleukin-1beta modulates proinflammatory cytokine production in human epithelial cells. Infect Immun.

[27] Hosokawa Y, Hosokawa I, Ozaki K, Matsuo T (2017). IL-27 modulates chemokine production in TNF-alpha -stimulated human Oral epithelial cells. Cell Physiol Biochem.

[28] Finoti LS, Nepomuceno R, Pigossi SC, Corbi SC, Secolin R, Scarel-Caminaga RM (2017). Association between interleukin-8 levels and chronic periodontal disease: a PRISMA-compliant systematic review and meta-analysis. Medicine (Baltimore).

[29] Gamonal J, Acevedo A, Bascones A, Jorge O, Silva A (2000). Levels of interleukin-1 beta, −8, and −10 and RANTES in gingival crevicular fluid and cell populations in adult periodontitis patients and the effect of periodontal treatment. J Periodontol.

[30] Belstrom D, Damgaard C, Kononen E, Gursoy M, Holmstrup P, Gursoy UK (2017). Salivary cytokine levels in early gingival inflammation. J Oral Microbiol.

[31] Deinzer R, Weik U, Kolb-Bachofen V, Herforth A (2007). Comparison of experimental gingivitis with persistent gingivitis: differences in clinical parameters and cytokine concentrations. J Periodontal Res.

[32] Schueller K, Riva A, Pfeiffer S, Berry D, Somoza V (2017). Members of the Oral microbiota are associated with IL-8 release by gingival epithelial cells in healthy individuals. Front Microbiol.

[33] Krisanaprakornkit S, Kimball JR, Weinberg A, Darveau RP, Bainbridge BW, Dale BA (2000). Inducible expression of human beta-defensin 2 by Fusobacterium nucleatum in oral epithelial cells: multiple signaling pathways and role of commensal bacteria in innate immunity and the epithelial barrier. Infect Immun.

[34] Han YW (2015). Fusobacterium nucleatum: a commensal-turned pathogen. Curr Opin Microbiol.

[35] Brennan CA, Garrett WS (2019). Fusobacterium nucleatum - symbiont, opportunist and oncobacterium. Nat Rev Microbiol.

[36] Jorth P, Turner KH, Gumus P, Nizam N, Buduneli N, Whiteley M (2014). Metatranscriptomics of the human oral microbiome during health and disease. MBio.

[37] Ximenez-Fyvie LA, Haffajee AD, Socransky SS (2000). Comparison of the microbiota of supra- and subgingival plaque in health and periodontitis. J Clin Periodontol.

[38] Park OJ, Yi H, Jeon JH (2015). Pyrosequencing analysis of subgingival microbiota in distinct periodontal conditions. J Dent Res.

[39] Turner MD, Nedjai B, Hurst T, Pennington DJ (1843). Cytokines and chemokines: at the crossroads of cell signalling and inflammatory disease. Bba-Mol Cell Res.

[40] Schaumann Teresa, Kraus Dominik, Winter Jochen, Wolf Michael, Deschner James, Jäger Andreas (2013). Potential Immune Modularly Role of Glycine in Oral Gingival Inflammation. Clinical and Developmental Immunology.

[41] Jo AR, Baek KJ, Shin JE, Choi Y (2014). Mechanisms of IL-8 suppression by Treponema denticola in gingival epithelial cells. Immunol Cell Biol.

[42] Dyminska L (2015). Imidazopyridines as a source of biological activity and their pharmacological potentials-infrared and Raman spectroscopic evidence of their content in pharmaceuticals and plant materials. Bioorgan Med Chem.

[43] Bickel M (1993). The role of interleukin-8 in inflammation and mechanisms of regulation. J Periodontol.

[44] Darveau RP (2009). The Oral microbial Consortium's interaction with the periodontal innate defense system. DNA Cell Biol.

[45] Agace W, Hedges S, Andersson U, Andersson J, Ceska M, Svanborg C (1993). Selective cytokine production by epithelial cells following exposure to Escherichia coli. Infect Immun.

[46] Yumoto H, Nakae H, Fujinaka K, Ebisu S, Matsuo T (1999). Interleukin-6 (IL-6) and IL-8 are induced in human oral epithelial cells in response to exposure to periodontopathic Eikenella corrodens. Infect Immun.

[47] Graves DT, Cochran D (2003). The contribution of interleukin-1 and tumor necrosis factor to periodontal tissue destruction. J Periodontol.

[48] Gomes FI, Aragao MG, Barbosa FC, Bezerra MM, de Paulo Teixeira Pinto V, Chaves HV (2016). Inflammatory Cytokines Interleukin-1beta and Tumour Necrosis Factor-alpha - Novel Biomarkers for the Detection of Periodontal Diseases: a Literature Review. J Oral Maxillofac Res.

[49] Huang GT, Kim D, Lee JK, Kuramitsu HK, Haake SK (2001). Interleukin-8 and intercellular adhesion molecule 1 regulation in oral epithelial cells by selected periodontal bacteria: multiple effects of Porphyromonas gingivalis via antagonistic mechanisms. Infect Immun.

